# A randomized controlled trial of stapled versus ultrasonic transection in distal pancreatectomy

**DOI:** 10.1007/s00464-021-08724-3

**Published:** 2021-09-13

**Authors:** Luca Landoni, Matteo De Pastena, Martina Fontana, Giuseppe Malleo, Alessandro Esposito, Luca Casetti, Giovanni Marchegiani, Massimiliano Tuveri, Salvatore Paiella, Antonio Pea, Marco Ramera, Alex Borin, Alessandro Giardino, Isabella Frigerio, Roberto Girelli, Claudio Bassi, Giovanni Butturini, Roberto Salvia

**Affiliations:** 1 Unit of General and Pancreatic Surgery, University of Verona Hospital Trust, Verona, Italy; 2 Unit of HPB Surgery, Pederzoli Hospital, Peschiera del Garda, Italy; 3 Unit of General and Pancreatic Surgery, G.B. Rossi Hospital, University of Verona – DSCOMI, P.Le Scuro 10, 37134 Verona, Italy

**Keywords:** Distal pancreatectomy, Pancreatic fistula, Stapler, Ultrasonic dissection

## Abstract

**Background:**

The pancreatic transection method during distal pancreatectomy is thought to influence postoperative fistula rates. Yet, the optimal technique for minimizing fistula occurrence is still unclear. The present randomized controlled trial compared stapled versus ultrasonic transection in elective distal pancreatectomy.

**Methods:**

Patients undergoing distal pancreatectomy from July 2018 to July 2020 at two high-volume institutions were considered for inclusion. Exclusion criteria were contiguous organ resection and a parenchymal thickness > 17 mm on intraoperative ultrasound. Eligible patients were randomized in a 1:1 ratio to stapled transection (Endo GIA Reinforced Reload with Tri-Staple Technology®) or ultrasonic transection (Harmonic Focus® + or Harmonic Ace® + shears). The primary endpoint was postoperative pancreatic fistula. Secondary endpoints included overall complications, abdominal collections, and length of hospital stay.

**Results:**

Overall, 72 patients were randomized in the stapled transection arm and 73 patients in the ultrasonic transection arm. Postoperative pancreatic fistula occurred in 23 patients (16%), with a comparable incidence between groups (12% in stapled transection versus 19% in ultrasonic dissection arm, *p* = 0.191). Overall complications did not differ substantially (35% in stapled transection versus 44% in ultrasonic transection arm, *p* = 0.170). There was an increased incidence of abdominal collections in the ultrasonic dissection group (32% versus 14%, *p* = 0.009), yet the need for percutaneous drain did not differ between randomization arms (*p* = 0.169). The median length of stay was 8 days in both groups (*p* = 0.880). Intraoperative blood transfusion was the only factor independently associated with postoperative pancreatic fistula on logistic regression analysis (OR 4.8, 95% CI 1.2–20.0, *p* = 0.032).

**Conclusion:**

The present randomized controlled trial of stapled versus ultrasonic transection in elective distal pancreatectomy demonstrated no significant difference in postoperative pancreatic fistula rates and no substantial clinical impact on other secondary endpoints.

## Introduction

Most of the research endeavors targeting risk factors for postoperative fistula (POPF) after distal pancreatectomy (DP) focused on the pancreatic transection method, a modifiable variable with the potential for improving fistula rates [1, 2]. The proposed technical variants included sharp transection with handsewn closure (using mattress or fish-mouth sutures), stapled transection, transection with energy-based devices (diathermy, ultrasonic devices, with or without ligation of the main pancreatic duct), or even anastomosis of the pancreatic stump to a Roux-en-Y jejunal limb or as a pancreaticogastrostomy [3–7]. Furthermore, the use of additional biologic sealants or stump reinforcement with an omental or falciform ligament patch have been investigated with mixed results [8, 9]. Remarkably, none of these techniques have demonstrated a clear superiority over the others in randomized controlled trials [10, 11]. Staplers and energy-based devices have been increasingly adopted in the last decade because of the more frequent use of minimally invasive approaches and the easy, fast, and reproducible mechanism of action. Recently a new type of triple-row stapler reinforced with a preloaded bioabsorbable polyglycolic acid (PGA) felt has been marketed, with preliminary data showing a decrease in the incidence and severity of POPF compared with the standard stapler and with ultrasonic devices, provided a pancreatic thickness < 17 mm [12–14]. In a recent retrospective, propensity-score matched analysis of 184 patients we suggested that the use of the triple-row reinforced stapler was associated with a sharp reduction of POPF rates relative to the ultrasonic dissector group (12% versus 40%) [15]. Under these premises, we sought to evaluate in a randomized trial whether parenchymal transection using the triple-row reinforced stapler decreases the incidence of POPF following DP compared with ultrasonic transection.

## Methods

### Study design and participants

**Fig. 1:**
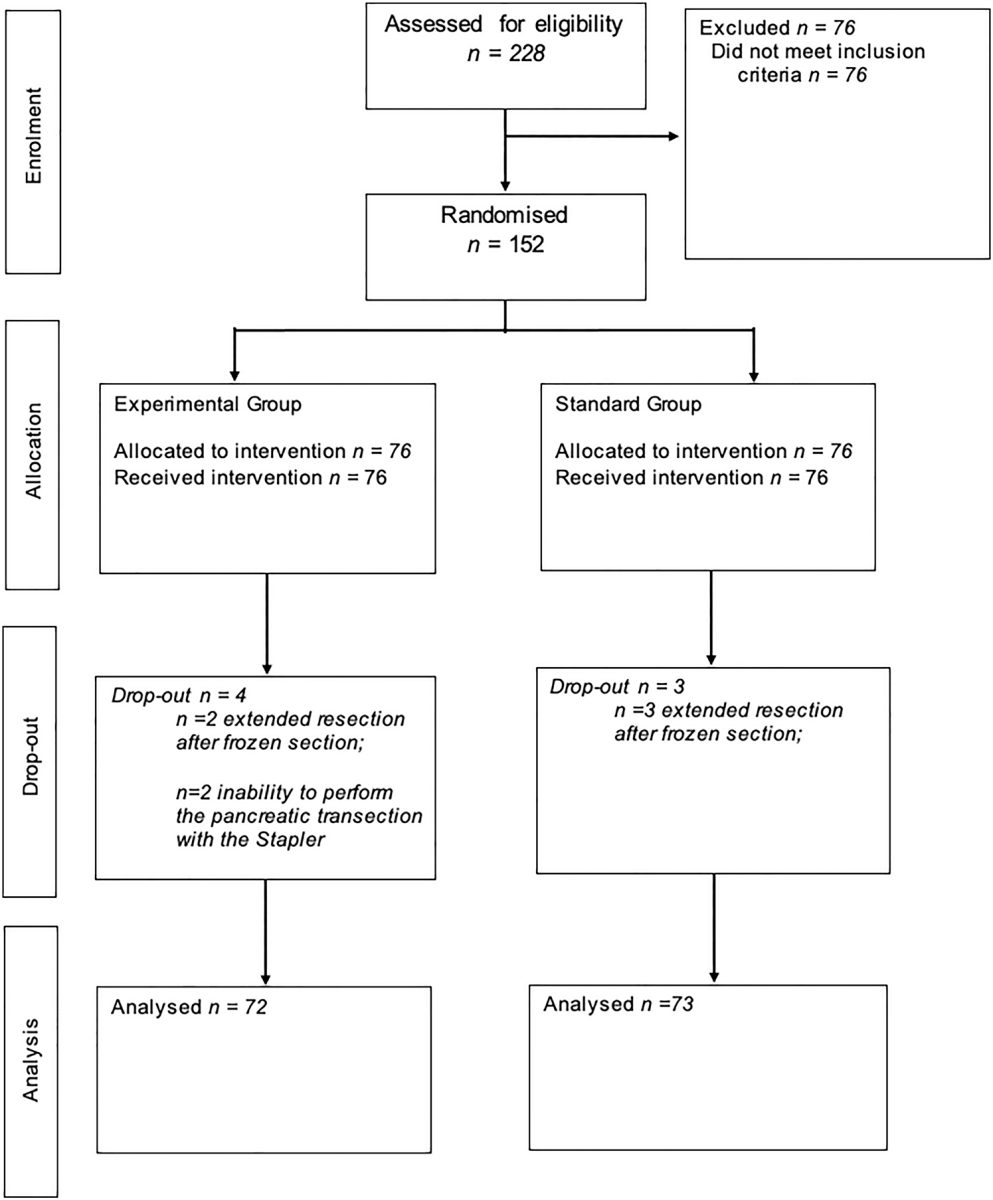
The CONSORT flowchart

This study is a bicentric, patient-blinded, randomized clinical trial conducted from July 2018 to July 2020 at the Unit of General and Pancreatic Surgery, University of Verona Hospital Trust, Verona, Italy; and the Unit of HPB Surgery, Ospedale Pederzoli, Peschiera del Garda, Italy. The study protocol was approved by the Ethics Committee of the Provinces of Verona and Rovigo (#1664CESC) and registered at ClinicalTrial.gov (NCT03880773). The trial was performed in accordance with the good clinical practice guidelines, the principles of the Declaration of Helsinki, and the Consolidated Standards of Reporting Trials (CONSORT) Guidelines [16]. Patients between the ages of 18 and 80 with any indication for elective DP were eligible for inclusion. All eligible patients provided written informed consent at the time of hospital admission. The CONSORT flowchart is reported in Fig. 1.

### Randomization and masking

The randomization process was as follows: on intraoperative exploration, patients were excluded if an extended DP was needed. This involved a posterior Radical antegrade modular pancreatosplenectomy (RAMPS) for left adrenal/kidney infiltration or a synchronous arterial resection (celiac trunk or hepatic artery) or an associated bowel resection. Synchronous venous resection was not an exclusion criterion. In eligible patients, pancreatic thickness was measured at the point of parenchymal transection via intraoperative ultrasound. Only patients with a parenchymal thickness < 17 mm were enrolled in the trial and randomized by telephone in a 1:1 ratio using a computer-generated randomization list kept by independent data managers at the coordinating center (Unit of General and Pancreatic Surgery, University of Verona Hospital Trust) and concealed to the investigators. Patients were blinded to the arm allocation during the postoperative course. The 17-mm cutoff was used to avoid staple closure failure or parenchymal crushing, according to previous evidence [12, 17]. Post-randomization drop-out occurred in the instance of positive transection margin on frozen section analysis, requiring further resection up to total pancreatectomy.

### Procedures

DP were performed by specialized pancreatic surgeons who completed the learning curve and had a personal annual caseload exceeding 50 major pancreatic resections and had completed the learning curve for both open and minimally invasive DP. All surgeons were familiar with both stump management techniques used in this trial. DP was performed either via laparotomy or minimally invasive approaches (laparoscopic or robot-assisted), with or without spleen preservation [18–20]. The level of pancreatic transection at the neck, body, or tail, depended on the nature and the location of the lesion. Stapled transection was performed using an Endo GIA Reinforced Reload with Tri-Staple Technology® (COVIDIEN, North Haven, CT, USA). Either a purple (3 mm) or black (4 mm) cartridge was employed according to the single surgeon’s preference. A gradual compression was applied for 2–3 min, the stapler was then fired and slowly released after transection. Ultrasonic transection was performed using the Harmonic Focus® + Shears (open surgery) or the Harmonic Ace® + Shears (minimally invasive surgery), HARMONIC, Johnson & Johnson Medical, Ethicon, Tokyo, Japan. Ultrasonic technology uses high-frequency mechanical energy that cuts by cavitational fragmentation and simultaneously seals tissues by coaptive coagulation [21]. The pancreas was transected at the lowest vibration level, no additional sutures were placed in the pancreatic stump or the main pancreatic duct. The Institutional policy regarding the intraoperative blood transfusion is very strict, with transfusions being indicated for Hb levels < 8 mg/dL or for hemodynamic instability. In both arms an easy-flow drain was placed in the proximity of pancreatic stump. Postoperative drain management was described elsewhere and was standardized across the participating institutions [22].

### Outcomes

The primary endpoint was the incidence of POPF as defined by the International Study Group of Pancreatic Surgery [23]. Secondary endpoints were any complications, classified according to the Clavien–Dindo score [24], major complications, defined as Clavien–Dindo grade III or higher, abdominal collections, delayed gastric emptying (DGE) and post-pancreatectomy hemorrhage (PPH), classified according to the International Study Group on Pancreatic Surgery definitions [25, 26], postoperative hospital stay (including readmission), and 90-day mortality. Follow-up visits were carried out at 30 and 90 days from the index operation.

### Statistical analysis

The study was designed hypothesizing that stapled transection was superior to ultrasonic transection. The sample size was calculated based on previously published institutional retrospective data reporting a 40% and 12% POPF rates following ultrasonic and stapled transection, respectively [15]. Assuming a 20% delta in the prospective trial, at a 5% alpha and 80% power (1-beta), the required sample size was 138 patients (69 per arm) according to the continuity corrected Z-Test with unpooled variance. Adjustment for post-randomization drop-out was made expecting a 10% rate of transection margin positivity on frozen section analysis, leading to a total sample size of 152 patients (76 per arm). Demographic and clinical characteristics were age, gender, American Society of Anesthesiologists (ASA) score, body mass index (BMI, kg/m2) categorized based on WHO classification [27], diabetes mellitus, age-adjusted Charlson comorbidity index score [28], chronic steroid therapy, neoadjuvant chemotherapy. Surgical variables included operative approach, conversion from minimally invasive to open approach, splenectomy, pancreatic gland thickness measured by intraoperative ultrasound at the point of transection, transection level categorized into gastroduodenal artery level, pancreatic neck, and left border of the aorta or more distal, vascular venous resection, intraoperative blood loss (mL), and operating time (minutes). In the stapled transection arm, the compression ratio (defined as the pancreas thickness divided by the closed length of the stapler), and the height difference (defined as the difference between the pancreatic thickness and the closed length of the stapler) were calculated [12, 29]. The values of closed length were defined per the manufacturer specifications.

Continuous variables were expressed as means with standard deviation or medians with interquartile range (IQR) and compared using *t*-test or Mann–Whitney test, as appropriate. Categorical variables were expressed as absolute numbers and percentages and compared using chi-square test or Fisher’s exact test. All tests were two-tailed. Binary logistic regression analysis was performed to investigate factors associated with POPF. Factors with a *p*-value < 0.1 on univariable analysis were entered in the model. Data are presented with odds ratios and 95% confidence intervals. A *p*-value < 0.05 was considered statistically significant. Data were analyzed using the Statistical Package for Social Sciences software, v25.0 for Windows (SPSS, Inc, Chicago, IL, USA).

## Results

### General characteristics

A total of 152 patients met the inclusion criteria and were randomized (Fig. 1). Due to a positive transection margin on frozen section analysis requiring further resection, seven patients were excluded post-randomization. Therefore, the final population comprised 72 patients in the stapled transection arm and 73 patients in the ultrasonic transection arm. The baseline characteristics by randomization arm are outlined in Table 1. The median pancreatic thickness measured intraoperatively at the transection level was 12 mm in both groups.

**Table 1:** Demographic, intraoperative, and pathological data

Study population *n* = 145			
	Total *n*° (%)	Stapled transection 72 (50%)	Ultrasonic transection 73 (50%)
Age (years, IQR)	60 [50–70]	62 [50–70]	60 [50–69]
Gender (Female)	87 (60)	48 (67)	39 (53)
BMI (Kg/m^2^, IQR)	25 [22–27]	24 [21–27]	25 [22–28]
Diabetes	24 (17)	13 (18)	11 (15)
ASA score ≥ III	18 (12)	8 (11)	10 (14)
Charlson Age > 4	48 (33)	25 (35)	23 (32)
Neoadjuvant therapy	31 (21)	15 (21)	16 (22)
Minimally invasive	59 (41)	29 (40)	30 (41)
Conversion^**#**^	2 (3)	0 (0)	2 (7)
Spleen preservation	24 (17)	10 (14)	14 (19)
Vascular resection	4 (3)	0 (0)	4 (6)
Transection level			
Pancreatic neck	104 (72)	50 (69)	54 (74)
GDA level	3 (2)	3 (4)	0 (0)
Left aortic border	38 (26)	19 (26)	19 (26)
IOUS thickness (mm, IQR)	12 [10–14]	12 [10–14]	12 [10–15]
Duration of Surgery (minutes, IQR)	251 [201–334]	246 [201–321]	257 [202–335]
EBL (cc, IQR)	100 [50–300]	100 [100–300]	150 [50–300]
Blood transfusion	11 (8)	4 (6)	7 (10)
Pathology, No. (%)			
PDAC	54 (37)	32 (44)	22 (30)
pNET	39 (27)	17 (24)	22 (30)
IPMN	8 (6)	4 (6)	4 (6)
MCN/SCN	30 (20)	16 (22)	14 (19)
SPN	6 (4)	2 (3)	4 (6)
Other	8 (6)	1 (1)	7 (9)

^#^ Referred to minimally invasive procedures

*BMI* body mass index, *ASA* American society of Anesthesiology, *GDA* gastroduodenal artery, *IOUS* intraoperative ultrasound, *EBL* estimated blood loss, *PDAC* pancreatic ductal adenocarcinoma, *pNET* pancreatic neuroendocrine tumor, *IPMN* intraductal papillary mucinous neoplasm, *MCN* mucinous cystic neoplasm, *SCN* serous cystic neoplasm, *SPN* solid pseudopapillary neoplasm

### Primary endpoint

Overall, 23 patients (16%) developed POPF (Table 2). There were 19 grade B (14%) and 4 grade C fistulas (2%). The incidence of POPF was similar between groups (12% in stapled transection versus 19% in ultrasonic dissection, *p* = 0.191). Biochemical leak (BL) occurred in 42 patients (29%), 21 patients in each arm (*p* = 0.552).

**Table 2:** Postoperative data

Study population *n* = 145				
	Total N (%)	Stapled transection 72 (50%)	Ultrasonic transection 73 (50%)	*p*-value
Any complication	57 (39)	25 (35)	32 (44)	0.170
POPF	23 (16)	9 (12)	14 (19)	0.191
Grade B	19 (14)	8 (12)	11 (16)	
Grade C	4 (2)	1 (1)	3 (4)	
Biochemical leak	42 (29)	21 (29)	21 (29)	0.552
Abdominal collection	33 (23)	10 (14)	23 (32)	**0.009**
Percutaneous drain	10 (7)	3 (4)	7 (10)	0.169
DGE	4 (3)	3 (4)	1 (1)	0.305
PPH	11 (8)	3 (4)	8 (11)	0.109
ICU Admission	17 (12)	7 (9)	10 (13)	0.314
Clavien–Dindo ≥ 3	19 (13)	6 (8)	13 (18)	0.074
Length of stay (days, IQR)	8 [6–13]	8 [6–13]	8 [6–12]	0.880
Reoperation	5 (3)	2 (3)	3 (4)	0.507
Readmission	14 (10)	4 (6)	10 (14)	0.083
Mortality	1 (1)	0 (0)	1 (1)	0.500

Bold value indicates statistical difference (*p*-value < 0.05)

*POPF* postoperative pancreatic fistula, *DGE* delayed gastric empty, *PPH* Post pancreatectomy hemorrhage, *ICU* intensive care unit

### Secondary endpoints

Table 2 shows the postoperative outcomes. In all, 57 patients (39%) had any complication, without differences between groups (35% in stapled transection versus 44% in ultrasonic transection, *p* = 0.170). There was an increased incidence of abdominal collections in the ultrasonic dissection group (32% versus 14%, *p* = 0.009). In both groups, one-third of patients with POPF required a percutaneous drain (*p* = 0.169). Five patients (3%) underwent reoperation, mostly for a hemorrhage (three of five patients), while the other two of five patients undergoing re-operation presented with sepsis due to infected POPF. There was one postoperative death in the ultrasonic dissection group. This patient died on postoperative day four of a sudden aortic arch dissection that was confirmed on autopsy. The median length of stay was similar between groups (8 days, *p* = 0.880).

Univariable analysis of factors associated with POPF, shown in Table 3, revealed a significant association with BMI, pancreas transection level, and intraoperative blood transfusion. The mean intraoperative estimated blood loss of transfused patients was 650 cc. Only in two cases there was massive intraoperative bleeding (> 2000 cc). In the stapled transection group, the compression rate and the height difference were not correlated with POPF (*p* = 0.362 and *p* = 0.979, respectively). Intraoperative blood transfusion was the only factor independently associated with POPF (OR 4.8, 95% CI 1.2–20, *p* = 0.032) on logistic regression analysis (Table 4).

**Table 3:** Univariable analysis of factors associated with POPF

Study population *n* = 145			
	POPF 23 (16%)	No POPF 122 (84%)	*p*-value
Age (years, IQR)	62 [55–71]	60 [50–69]	0.564
Sex (Female)	11 (48%)	76 (62%)	0.143
BMI (Kg/m^2^, IQR)	26 [25–29]	24 [21–27]	0.013
Diabetes	2 (9%)	22 (18%)	0.218
ASA score ≥ III	3 (13%)	15 (12%)	0.573
Charlson age > 4	8 (35%)	40 (33%)	0.514
Neoadjuvant therapy	5 (22%)	26 (21%)	0.577
Thickness neck (mm, IQR)	14 [12–15]	11 [9–13]	** < 0.001**
Duct size (mm, IQR)	2 [1–3]	1 [1, 2]	0.482
Minimally invasive DP	12 (52%)	47 (39%)	0.161
Spleen preservation	4 (17%)	20 (16%)	0.555
Vascular resection	1 (4%)	3 (3%)	0.503
Transection level			**0.040**
Pancreatic neck	14 (13%)	90 (87%)	
GDA level	2 (67%)	1 (33%)	
Left aortic border	7 (18%)	31 (82%)	
IOUS thickness (mm, IQR)	13 [11–15]	12 [10–14]	0.307
Compression rate^#^ (mm, SD)	3,5 ± 0,5	3,4 ± 0,6	0.362
Height difference^#^ (mm, SD)	8,3 ± 1,8	8,3 ± 2,2	0.979
Duration of Surgery (minutes, IQR)	293 [216–378]	246 [201–321]	0.126
EBL (cc, IQR)	200 [75–300]	100 [50–300]	0.399
Blood transfusion	5 (22%)	6 (5%)	**0.016**
Pathology PDAC	10 (19%)	13 (14%)	0.326

Bold values indicate statistical difference (*p*-value < 0.05)

*BMI* body mass index, *ASA* American society of Anesthesiology, *GDA* gastroduodenal artery, *IOUS* intraoperative ultrasound, *EBL* estimated blood loss, *PDAC* pancreatic ductal adenocarcinoma

^#^Related only to the reinforced stapler group

**Table 4:** Logistic regression of factors associated with POPF

Study population *n* = 145			
	POPF	*p*-value	OR (CI 95%)
BMI (Kg/m^2^)			
< 24,9 kg/m^2^	7 (10%)	1	\
25–29,9 kg/m^2^	11 (20%)	0.209	1.9 (0.6–5.8)
> 30 kg/m^2^	5 (29%)	0.924	1 (0.4–2.3)
Transection level			
Pancreatic neck	14 (13%)	1	\
GDA level	2 (67%)	0.357	1.4 (0.6–3.4)
Left aortic border	7 (18%)	0.114	0.2 (0.3–1.5)
Blood transfusion			
No		1	\
Yes	5 (46%)	**0.032**	4.8 (1.2–20)

Bold value indicates statistical difference (*p*-value < 0.05)

*BMI* body mass index, *GDA* gastroduodenal artery, *POPF* postoperative pancreatic fistula

## Discussion

The present randomized clinical trial of stapled versus ultrasonic transection in DP demonstrated no significant difference in POPF rates. Analysis of secondary outcomes revealed a greater incidence of abdominal collections in the ultrasonic dissection arm, although the need for percutaneous drains was comparable between groups. POPF therefore remains a clinically relevant and unsolved issue for patients undergoing elective DP, with a formation process likely independent on the surgical technique adopted for resection and closure of the pancreatic remnant. Our findings indeed resonate with previously published randomized controlled trials that did not identify an optimal transection method able to decrease POPF [30].

To the best of our knowledge, this is the first randomized trial of a triple-row stapler reinforced with a preloaded PGA felt. Previous studies had already shown that wrapping the pancreatic stump with a PGA mesh decreased the rate of POPF [31, 32], and triple-row stapler had been associated with less POPF compared with the double-row staplers [33]. The Endo GIA Reinforced Reload with Tri-Staple Technology® has been available at the authors’ institution since its introduction into the market and has been employed at the surgeon’s discretion for parenchymal transection in DP. A retrospective propensity-matched analysis comparing surgical outcomes with ultrasonic dissection (HARMONIC® Focus + or Ace +) showed a significantly decreased rate of POPF in the reinforced Tri-Staple group (12% versus 40%), constituting the backbone for the present trial [15]. As suggested by earlier studies, patients with a parenchymal thickness > 17 mm were excluded because of a very high incidence of POPF that was independent on the type of cartridge, because of stapler closure failure of parenchymal crushing [12]. In patients who were randomized to stapled transection we gradually compressed the pancreas with the stapler for about 2–3 min, then divided the parenchyma and released the device slowly. This has been shown to help avoiding the development of POPF [34]. Nonetheless, the choice of the stapler cartridge was left at the single surgeon’s discretion. While cartridges with closed length < 15 mm (i.e., purple) have been shown to be particularly suitable for thin pancreata (< 12 mm), in thicker glands a longer staple height has been recommended (i.e., black) although no particular cartridge has proven to outperform the others.

In the ultrasonic dissection arm, the pancreas was transected and simultaneously sealed by coaptive coagulation at the lowest vibration level. Experimental studies proved that the lateral thermal spread is limited to 0–2 mm beyond the tissue grasped within the forceps of the device [35]. The decreased propensity for collateral thermal damage is an important putative advantage of the Harmonic scalpel, particularly when compared with other energy devices such as monopolar and bipolar diathermy, which are commonly used for pancreatic transection in DP [30]. However, an independent association between ultrasonic transection and a slower POPF healing has been suggested by our group [36]. Whether this depends on thermal effects has to be fully elucidated.

Analysis of factors associated with POPF suggested that BMI and the anatomic transection level play an integral role to the process. BMI is indeed a surrogate of fatty infiltration that has been shown to correlate with a complicated clinical course [37]. Even the transection level has been widely linked to POPF, because the pancreas shape and thickness are different at the gastroduodenal artery level, at the neck, or in the body and tail [8, 11]. Nonetheless, only intraoperative blood transfusion was independently associated with POPF on multivariable analysis. This is in accordance with a recent systematic review and meta-analysis, and might serve as a surrogate parameter for pancreatic stump ischemia [38]. Taken together, these results emphasize the need for perioperative composite scores to predict high-risk scenarios and help establishing individualized prevention and mitigation strategies. While these tools have been derived and successfully validated in pancreatoduodenectomy [39], previous efforts in large, multi-institutional DP series have proven elusive [40].

The study has some limitations. First, sub-analysis of stapler cartridges was not done. The liberal use of purple or black cartridges with PGA reinforcement possibly introduced a bias, despite the compression rate and the height difference were not associated with POPF. Another limitation could be the difference in the anatomic point of parenchymal transection. Nonetheless, the point of transection was dictated by the underlying pathology, with parenchyma-sparing procedures being favored in the context of benign to low-grade neoplasms, and this parameter did not result to be a risk factor at the adjusted analysis.

In conclusion, the present randomized controlled trial of stapled transection using a PGA-reinforced triple-row stapler versus ultrasonic transection with HARMONIC® energy devices in elective DP demonstrated no significant difference in POPF rates and no substantial clinical impact on other secondary endpoints. Therefore, the optimal technique for the management of pancreatic stump in resection of the left pancreas remains unclear and warrants further investigation.
